# Multiple Isolated Transcription Factors Act as Switches and Contribute to Species Uniqueness

**DOI:** 10.3390/genes11101148

**Published:** 2020-09-29

**Authors:** Xin-Wei Zhao, Hirohisa Kishino

**Affiliations:** Graduate School of Agricultural and Life Sciences, The University of Tokyo, Tokyo 113-8657, Japan; kishino@g.ecc.u-tokyo.ac.jp

**Keywords:** transcription factor, regulatory network, mammal

## Abstract

Mammals have variable numbers (1300–2000) of transcription factors (TFs), but the reasons for this large variation are unclear. To investigate general TF patterns, we de novo identified 156,906 TFs from 96 mammalian species. We identified more than 500 human isolated TFs that are rarely reported in human TF-to-TF networks. Mutations in the genes of these TFs were less lethal than those of connected TFs. Consequently, these isolated TFs are more tolerant of changes and have become unique during speciation. They may also serve as a source of variation for TF evolution. Reconciliation of TF-family phylogenetic trees with a mammalian species tree revealed an average of 37.8% TF gains and 15.0% TF losses over 177 million years, which implies that isolated TFs are pervasive in mammals. Compared with non-TF interacting genes, TF-interacting genes have unique TF profiles and have higher expression levels in mice than in humans. Different expression levels of the same TF-interacting gene contribute to species-specific phenotypes. Formation and loss of isolated TFs enabling unique TF profiles may provide variable switches that adjust divergent expression profiles of target genes to generate species-specific phenotypes, thereby making species unique.

## 1. Introduction

Gene expression patterns vary among species—even among closely related species that share highly similar genomic sequences. These differences in gene expression and regulation are believed to be the major sources of species phenotypic variation and important factors in evolution [1].

For many years, mutations in TFs have been thought to be the least likely source of variation, mainly because they can be responsible for negative pleiotropic effects [2,3,4]. When a mutation arises in protein-coding regions of a transcriptional regulator, multiple target genes of the regulator are simultaneously affected, potentially causing large-scale detrimental effects [5]. Genetic perturbations of 304 human/mouse TF orthologs in mouse associate with phenotypes and many individual TF loci have strong GWAS signals for multiple diseases [6]. HOX TF genes play a key role in proper body pattern formation [7], while SRY, a TF gene, is important for sex determination [8]. In particular, C2H2 zinc finger proteins were found to diversify rapidly and to represent most of the rapidly evolving human TFs [9,10].

During the past decade, an ever-increasing number of hidden Markov models of DNA binding domains (DBDs) and the growing sensitivity of TF detection procedures based on these models have contributed to the expansion of TF databases [11,12]. Several animal TF databases have been established, such as animalTFDB3 [13], Riken mouse TFdb [14], FlyTF [15], TFCat [16], TFCONES [17], ITFP [18], and humanTFs [6]. These databases collectively contain variable numbers of TFs from different species. Scanning of these databases suggests that the number of non-orthologous TFs is significant. Recent research on C2H2 TF families has also revealed the variability of TFs, but the relative frequency and consequences of global variation remain largely unexplored.

Although the systematic mapping of protein–protein interaction (PPI) is far from complete, it enables developmental and disease mechanisms at the system level to be understood by associating the global topology and dynamic characteristics of the interactome network with known biological characteristics [19,20]. Orthologous human and mouse TFs show preserved TF–TF interactions in a TF-to-TF network [5]. In contrast, information regarding the effects of non-orthologous TFs on gene regulatory networks is still limited. TFs with only non-TF interactions are usually ignored in TF-to-TF networks because they lack TF–TF interactions and are considered non-conservative. Since orthologous TFs are shared by both species, they are expected to be the core elements of the regulatory networks. Species specificity may be generated by microevolution of these orthologous TFs or their downstream target genes in each species lineage or it may be generated by rewiring the transcription networks by acquisition of new TFs and loss of existing TFs in each lineage. Because the second scenario has been largely neglected, we attempted to characterize it in this paper. Some TF/protein interactions are less well documented; however, their conservation tends to be low and mutated TFs are likely to be lethal, so they are more likely to achieve lineage-specific adaptation (reviewed in [21]). What then, are the TFs with rare TF interactions in TF-to-TF networks? How do these TFs work to enable different numbers of TFs between species?

Based on the above findings, we identified such transcription factors, and they conformed to the speculated characteristics described in previous studies. We further investigated the origins, consequences, and underlying regulatory logic of TF evolution for this set of isolated TFs.

## 2. Materials and Methods

### 2.1. Mammalian TF Database Construction

Hidden Markov models allow us to detect DBDs of TFs based on protein structural information rather than sequence similarity. Using this method, many TF databases have recently been established [16,17,22]. To construct a mammalian database de novo, we used complete protein sequences from the genomes of 96 species archived at NCBI [23] (many species were newly included) and hidden Markov models of 66 DBDs obtained from Pfam [12]. We used HMMER version 3.1b [11] with an *E*-value threshold of 0.0001 to find all protein sequences belonging to each TF family. To remove redundancies, protein names were annotated, and only protein isoforms with the highest scores were retained; in addition, alternative splicing types were filtered out after TF detection. After removal of duplicates, we obtained 156,906 TF proteins, nearly all of which were mammalian TF proteins. In human, we obtained 1625 TFs. To check the output data of our TF proteins, we used the Pfam online tool [12] to annotate protein domains of human TF proteins. We identified 1585 human TF proteins <1 kilobase (the lower Pfam input DNA sequence-length limit), all of which produced the correct TF DBD annotation.

### 2.2. TF Formation and Loss Detection

Sequences of each TF family from the 96 mammalian species were pooled together and aligned using MAFFT7 [24] and MUSCLE3.8 [25]. The aligned datasets were imported into DAMBE5 [26], converted to MEGA format, and used to construct phylogenetic trees of mammalian TF families in MEGA6 [27]. Among them, 48 neighbor-joining trees of TF families had a small number of members and could be constructed. To detect historical events, a species tree (from TIMETREE [28]) was reconciled with each of the 48 phylogenetic trees of TF families using NOTUNG 2.9 [29]. Rearrangement was performed to fit the structure of the species tree and to detect formation and loss events. After recording formation and loss events, we calculated the percentage of change on each branch of the species phylogenetic tree.

### 2.3. Construction of Protein–Protein Interaction (PPI) Networks

Mice (*Mus musculus*) and rats (*Rattus norvegicus*) are closely related species that diverged 20.9 million years ago [28]. All differences between mouse and rat networks can be assumed to have arisen recently. We therefore used mouse and rat data to detect factors that affect network evolution. Humans and mice are more genetically and phenotypically diverged, and much research has been conducted on these two species. We thus looked for human and mouse phenotype and expression differences caused by network changes. Whole protein network data of humans, mice, and rats (from STRING [30]) were used to construct PPI networks for these species. Within each network, all interactions had confidence scores ≥ 0.4 (medium + high confidence). Global PPI networks for mice (19,505 nodes and 847,065 edges), rats (19,920 nodes and 1,099,355 edges), and humans (18,720 nodes and 782,253 edges) were then constructed. In human, 1555 TFs had TF interactions or non-TF interactions. To detect isolated TFs (TF with only non-TF interactions or disconnected from the main TF group), these 1555 human TF nodes and TF–TF interactions were used to construct the TF-to-TF network. STRING collects protein–protein interactions based on multiple types of evidence: co-expression, high-throughput laboratory experiments, previous knowledge in databases, genomic context predictions and automated text-mining. For our network construction, we adopted interactions when there was any evidence regarding the type of interaction. If there is noise in the database, our networks may include false positive interactions but the chance of false negatives is minimized to give reliable information on isolated TFs.

### 2.4. Functional Cartography of the Human PPI Network

Using a previously published functional cartography protocol [31], we characterized each gene in the human PPI network according to its within-module degree *z*-score (*z*) and participation coefficient (*p*). The within-module degree *z*-score of node *i*, $z_{i}$, was calculated as:

$$z_{i}=\frac{k_{i}-\;\bar{k_{s_{i}}}}{\sigma_{k_{s_{i}}}}$$

where $k_{i}$ is the number of links between node *i* and other nodes in its module, $k_{s_{i}}$ is k averaged over all nodes in $s_{i}$, and $\sigma_{k_{s_{i}}}$ is the standard deviation of k in $s_{i}$. The participation coefficient of node *i*, $p_{i}\;$, was calculated as:

$$p_{i}=\;1-\;\overset{N_{M}}{\underset{s=1}{\sum }}\;{\left(\frac{k_{is}}{k_{i}}\right)}^{2}$$

where $k_{is}$ is the number of links between node *i* and other nodes in module *s*, and $k_{i}$ is the total degree of node *i*.

Genes were classified into eight groups: (1) those with no experimental interactions, (2) ultra-peripheral nodes (*z* < 2.5 and *p* < 0.05), (3) peripheral nodes (*z* < 2.5 and 0.05 ≤ *p* < 0.625), (4) non-hub connector nodes (*z* < 2.5 and 0.625 ≤ *p* < 0.8), (5) non-hub kinless nodes (*z* < 2.5 and *p* ≥ 0.8), (6) provincial hubs (*z* ≥ 2.5 and *p* < 0.3), (7) connector hubs (*z* ≥ 2.5 and 0.3 ≤ *p* < 0.75), and (8) kinless hubs (*p* ≥ 0.75).

### 2.5. Negative Binomial Regression Analysis of the Effect of TF Membership Variation on Gene Expression

Gene expression data of 15,796 orthologous human and mouse genes in five organs (cerebellum, heart, kidney, liver, and testis) were retrieved [32,33]. After standardization of these data as transcripts per kilobase per million reads, similar average expression levels were observed in each organ between humans and mice. To analyze the effect of the variation in the membership of TF families on the expression of their interacting genes, all orthologous genes were separated into five types: (1) genes without TF interactions, (2) genes with orthologous TF interactions, (3) genes with interactions with human- and mouse-specific TFs, (4) genes with interactions with human-specific TFs absent in mice, and (5) genes with interactions with mouse-specific TFs absent in humans. For each species and organ, we estimated gene expression profiles by negative binomial regression:

$$\log E\left[\mathrm{expression}|\mathrm{gene type}=C_{k}\right]=\alpha +\beta_{k}$$

using glm.nb in the R package MASS [34,35]. In this equation, the coefficient $\beta_{k}$ is the log mean expression of other groups relative to the reference group (genes without TF interactions).

### 2.6. TF-GO Bipartite Graphs for Humans and Mice

Gene ontology (GO) data on human and mouse TFs were retrieved [36,37]. The intersection of every TF associated with a GO term was checked between humans and mice, and the proportion of intersecting TFs relative to the average number of TFs was obtained by local polynomial regression using loess in R [35,38].

### 2.7. Data Availability

Protein sequences: NCBI [23]; http://www.ncbi.nlm.nih.gov

DNA binding domain (DBD) models: Pfam [12]; https://pfam.xfam.org

Species tree: TIMETREE [28]; http://www.timetree.org

Protein interaction data: STRING [30]; https://string-db.org

Gene ontology data: (1) Gene Ontology Consortium [36], http://www.geneontology.org and (2) g:Profiler [37], https://biit.cs.ut.ee/gprofiler

Gene name data: DAVID [39]; https://david.ncifcrf.gov

Phenotypic data: MGI [40]; http://www.informatics.jax.org

TF data, phylogenetic trees, and other data related to our research: https://github.com/zhaoxinwei90/supplementary-data

## 3. Results

### 3.1. Isolated TFs in a Human TF-to-TF Network Often Have No Orthologs in Mouse

We constructed a PPI network of nearly all human genes and a TF-to-TF network based on the detected TF list from the whole gene network (see Materials and Methods). Interactions were found between 1555 of the 1625 human TFs in the PPI network. This means these 1555 TFs have been previously investigated and that interactions have been determined with other TFs or non-TFs. One-third (515) were isolated from the other 1040 TFs (no conserved co-expression, high-throughput laboratory experiments, previous knowledge in databases, genomic context predictions or automated text-mining interaction), but were connected with non-TF genes (Table 1, Supplementary Materials, Figure S1 and Table S1). Out of 1040 TFs in the large connected component of the network, 507 (48.8%) were lethal when mutated, and only 40 (3.8%) were not found in mice. In contrast, 26 (5.0%) of the 515 isolated TFs were lethal when mutated, and 189 (36.7%) were absent in mice. The average degree (number of connections) of the 515 isolated TFs in the human gene TF-to-TF network was 10.5 ± 8.8 (mean ± standard deviation), whereas TFs in the large connected component had an average degree of 77.9 ± 127.8. TFs having fewer documented interactions are less conserved and less lethal and are therefore more likely to enable lineage-specific adaptation (reviewed in [21]). Isolated TFs are consistent with the characteristics of this type of TF. Overall, TFs that are isolated in the TF-to-TF network generated TF number variation, and the human TFs absent in mice are more dispensable for TF–TF interactions. We additionally conducted a functional cartographic analysis [31] of all TF and non-TF genes in the human PPI network (Supplementary Materials, Figure S2). TFs were not at the core of the human PPI network, but were on the periphery, even compared with non-TF genes. This observation is consistent with the variable TF profile uncovered when non-orthologous TFs are also considered. However, TFs in the large connected component, which is enriched in orthologous TFs, are evolutionarily conserved. In human TF profile, 229 TFs are different when compared with mice.

Among the 229 TFs, 189 belong to isolated TFs. The isolated TFs are largely human-specific; they contribute most to TF profile differentiation, at least among human and mice.

### 3.2. TF Families Vary Greatly in Scale among Mammalian Species

**Figure 1:**
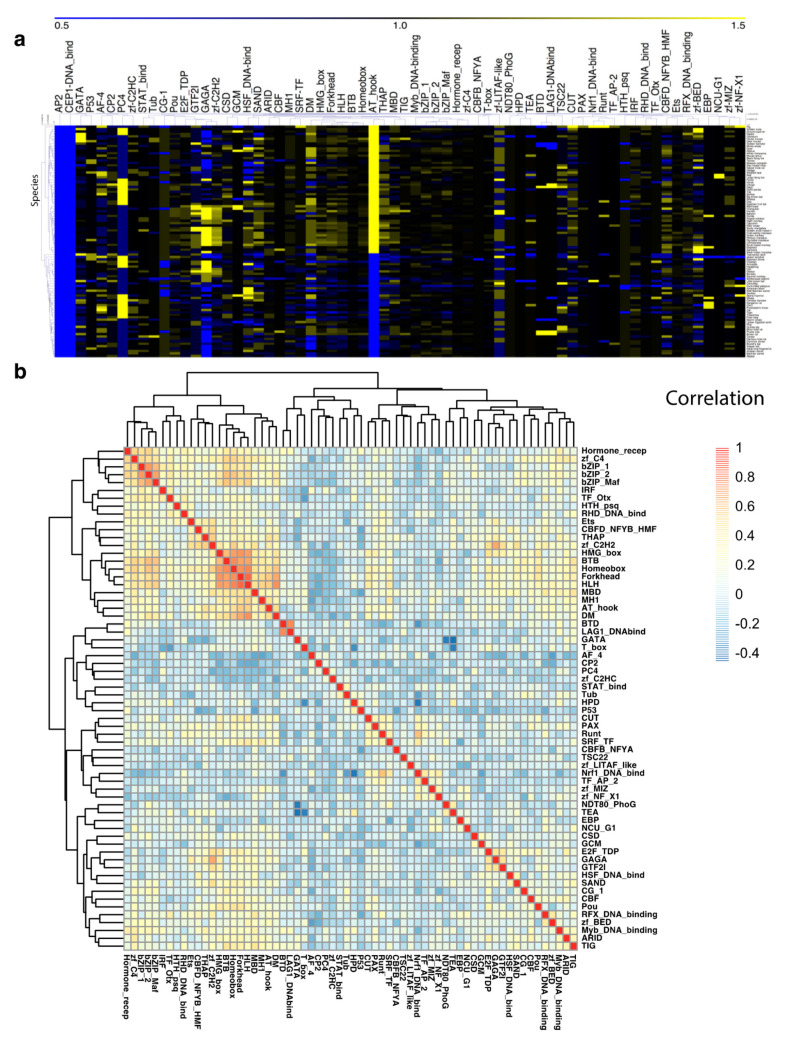
Variation in the number of transcription factors (TFs) within and among TF families. The dendrograms show the hierarchical relationships. (**a**) Variation in the number of TFs within each TF family. X-axis: TF family; y-axis: mammalian species. The average number of TFs in each TF family was standardized to 1 (black). The colors on the heat map represent the degree of TF number variation, where blue is low and yellow is high. (**b**) Correlation of TF number variation among TF families. The colors on the heat map represent the degree of correlation (blue, low; red, high).

TF families vary in scale because of gene duplication and loss, as well as the loss of DBDs. To examine variation in the membership of TF families, we de novo detected 156,906 putative TFs belonging to 66 TF families in 96 mammalian genomes (Supplementary Materials, Figure S3, Table S1). The total number of TFs varied substantially among species. For example, *Neotoma lepida* had 1337 TFs, whereas a closely related species, *Peromyscus maniculatus bairdii*, had 1628. Using a standardized number of each TF family as a control, we observed that variation in membership was also very widespread among these TF families (Figure 1a). We examined the correlation between TF families and found that 97.9% of TF family pairs (1973 out of 2016) were not strongly correlated (*r* < 0.5). This result indicates that number variations in each TF family tend to be independent of other families. In the TF-family correlation matrix and heatmap shown in Figure 1b, only three small clusters have members that are strongly correlated with one another: (1) bZIP_1, bZIP_2, and bZIP_Maf; (2) BTD and LAG1_DNAbind; and (3) HMG_box, BTB, Homeobox, Forkhead, and HLH. bZIP_1, bZIP_2, and bZIP_Maf are all present in 14 mouse TF genes, while BTD and LAG1_DNAbind are both located in two mouse TF genes. Two members of cluster 3, HMG_box and HLH, are both found in the gene encoding protein S9YBX2. In other words, these strong correlations mostly result from genes sharing multiple DBDs rather than the co-occurrence of gene duplications or losses.

**Figure 2:**
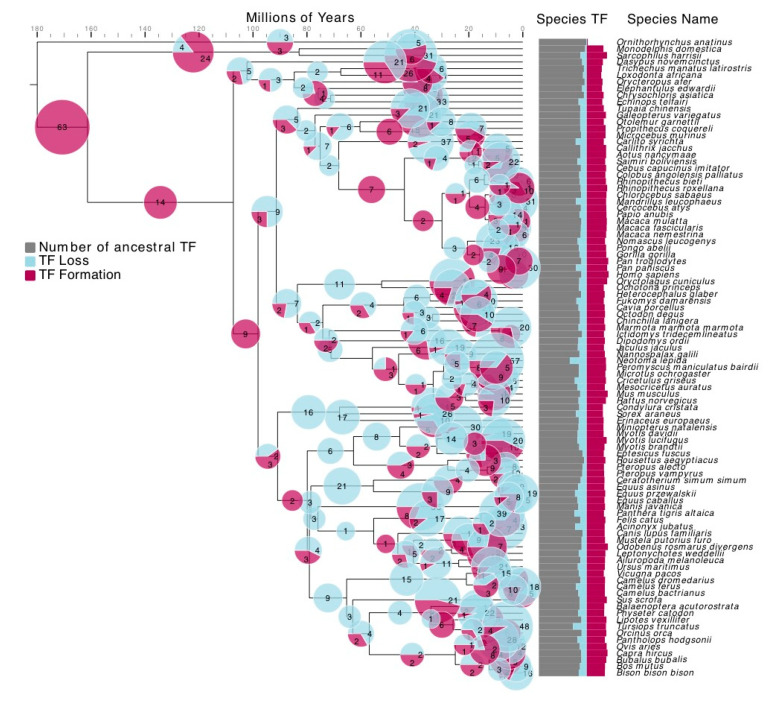
Atlas of formation and loss events in 48 transcription factor (TF) families from 96 mammalian species over 177 million years. The size of each pie chart is proportional to the number of TF gains and losses on each branch; light blue indicates TF loss events, and red indicates TF formation events. The bar chart displays the total number of TF gains (red) and losses (blue) in each species over 177 million years. The gray bars indicate ancestral TFs. Species tree is from TimeTree [28].

Large TF families, such as C2H2, have been found to rapidly diversify. Families with limited members are usually thought to be more conserved and are less researched. To reveal the detailed history of variation in TF family membership, phylogenetic trees of 48 small size TF families were reconciled with the mammalian species tree [28]. The membership of different TF families was found to have changed along nearly all branches of the mammalian species tree (Figure 2). Compared with the common mammalian ancestor, an average of 37.8% of the TFs of a mammalian species arose during its evolution, whereas 15.0% disappeared. This high level of turnover, more than half of the TFs of a species, indicates that TF families have generally undergone substantial alteration through isolated TFs. Unlike TF orthologs [5], these TF families as a whole are not as conserved as previously thought. TF formation and loss have occurred even more extensively along recent branches. These TF formation and loss events have shaped the unique TF profile of each species. Among 48 TF families (Supplementary Materials, Table S2), abundant gains and losses have taken place in families such as GATA and Forkhead. Members of the GATA TF family, which include more than 15% of all gained TFs, are inducers of the pluripotency reprogramming and may serve as important mediators of cell fate conversion [41]. The Forkhead TF family, which includes 14.5% of all lost TFs, regulates cell growth, proliferation, differentiation, and longevity [42]. The functional importance of TFs is therefore not dependent of evolutionary conservation. TF gains and losses have been prevalent during mammalian evolution. Since the software Notung only provides event numbers, we could not check the proteins that experienced the events in detail. To obtain a clear picture on the effect of TF losses, we focused on human and mice and conducted quantitative analysis. We will try to find better ways to apply quantitative analysis to whole mammal species in future research.

### 3.3. Genes Interacting with TFs in Humans and Mice Have Similar Expression Profiles but Are More Highly Expressed in Mice

Variation in the membership of TF families influences the PPI network. The formation of TFs adds new edges, while the loss of TF genes removes them. To determine the effect of DBD loss, we compared the global PPI networks of two closely related species, mice and rats. The mouse network contained 19,505 nodes and 847,065 edges, while the rat network consisted of 19,920 nodes and 1,099,355 edges. Within these networks, we focused on the TF subnetworks (1440 and 1288 TF genes in mice and rats, respectively) and their interacting genes. Without considering DBD loss, roughly the same numbers of orthologous TFs were found to interact, with a relative difference of 30.1 ± 22.3% (Supplementary Materials, Table S3). When DBD loss was considered, the relative difference in the number of interacting genes increased to 50.7 ± 27.9%. In general, a change in a DBD doubled the variation in the number of interacting genes.

**Figure 3:**
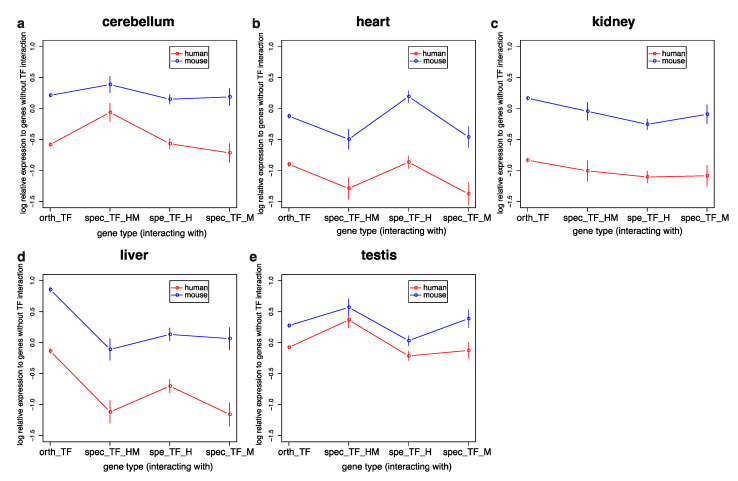
Log expression levels of transcription factor (TF)-interacting and non-interacting human and mouse orthologous genes. (**a**)Expression of genes in cerebellum. (**b**)Expression of genes in heart. (**c**)Expression of genes in kidney. (**d**)Expression of genes in liver. (**e**)Expression of genes in testis. Standard error bars are attached to the means. Orthologous genes were divided into four groups according to their interactions with TFs, namely, those that interact with orthologous TFs (orth_TF), human- and mouse-specific TFs (spec_TF_HM), human- but not mouse-specific TFs (spec_TF_H), and mouse- but not human-specific TFs (spec_TF_M).

Variation in TF-interacting genes among species may affect their expression profiles. Figure 3 shows the expression profiles of orthologous genes in humans and mice (Supplementary Materials, Table S4) relative to the expression of non-TF-interacting genes. Generally, the relative expression of TF interacting genes compared to non-TF interaction genes is higher in mice than in humans, although the difference is small in the testis. In the cerebellum and testis, genes interacting with human- and mouse-specific TFs have higher expression levels, especially in humans. In the heart, genes interacting with human-specific TFs have the highest expression, especially in mice. In the liver, genes interacting with orthologous TFs have the highest expression in both humans and mice. Variation in expression profiles is small in the kidney.

### 3.4. Loss of Human TFs in Mice Reveals Knockout-Phenotypes of Their Targets in Humans

Human-isolated TFs are enriched in the Cys2His2-zinc finger (C2H2-zf) TF family. To compare the effect of reducing their expression levels in humans and mice, we focused on the C2H2-zf-containing Krüppel-associated box (C2H2-KRAB) family, the largest individual genome-encoded transcriptional repressor family of higher organisms [43]. We surveyed the knock-out phenotypes of 672 C2H2-KRAB-interacting genes [40]. A total of 9827 mammalian phenotype terms were recorded (Supplementary Materials, Table S5). We then collected information on mouse C2H2-KRAB-interacting genes that do not participate in this interaction and that are known to be responsible for specific mammalian phenotypes (Table 2). We looked at the function of genes whose expression is regulated by human specific TFs and these human specific TFs also belong to C2H2-KRAB family. Because transcription factors can up-regulate or down-regulate the expression of target genes, and the C2H2-KRAB family is mainly down-regulated, thus the target genes of C2H2-KRAB are selected as extra criteria to keep the direction of regulation as consistent as possible. According to the knockout data of mouse genes, the phenotypic changes that may be caused by the down-regulation of target gene expression caused by the presence of transcription factors in humans are simulated. As demonstrated by C2H2-KRAB knock-out phenotypes in mice, morphological differences in the corresponding phenotype between humans and mice are due, at least partially, to the reduced expression levels of these interacting genes in humans relative to mice. For example, the “short tail” (vs. “long tail”) phenotype in knock-out mice is consistent with the absence of tails in humans, while “delayed tooth eruption” (vs. “continually growing teeth”) in knock-out mice is comparable to permanent teeth in humans. Other examples include hair and skin phenotypes. Although information about target genes of species-specific TFs is lacking, we also found a similar trend in TF to target-gene regulation. The human-specific TF, SHOX, activates the expression of its target gene, FGFR3 [44]. The absence of SHOX in mice may contribute to the lower expression of mouse FGFR3. In mice, a humanized FGFR3 gene leads to “short tail” phenotypes, whereas knock-out of FGFR3 causes “long-tail” phenotypes (Supplementary Materials, Table S6). These findings indicate that species-specific TFs are responsible for diverse target-gene expression because of altered regulatory interactions and that divergent expression of genes shapes species-specific phenotypes (Supplementary Materials, Figure S4).

The same logic can also be applied to pathways. Small animals, such as mice, have a high metabolic rate. The glycolytic pathway is the basic pathway that supports the metabolic demands of different organisms. The isolated TFs can modulate five connected genes (TPI1, NLK, ALDOA, PFKL, and PFKM) in the glycolytic pathway, possibly making these genes more plastically regulated (Supplementary Materials, Figure S5a). Only two TFs (ZNF224 and ZNF256) interacting with ALDOA in humans are absent in mice. ZNF224 represses transcription of the ALDOA gene, and ZNF256 is a transcriptional repressor [45]. Consequently, ALDOA has relatively lower expression in humans than in mice. In all five organs, expression of the ALDOA gene is nearly double in mice compared with that in humans (Supplementary Materials, Figure S5b). This result indicates that the pathway can also be affected by the evolution of isolated TFs.

### 3.5. Human and Mouse Biological Functions Are Regulated by Similar Numbers of TFs but Different TF Family Members

**Figure 4:**
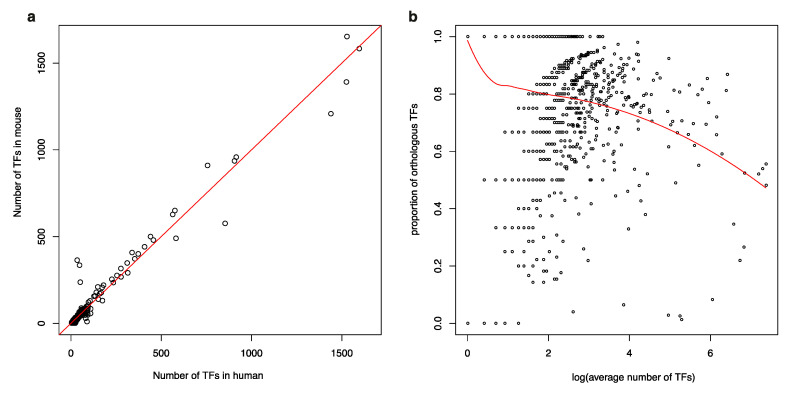
Shared and specific transcription factors (TFs) that regulate gene ontology (GO) terms in humans and mice. (**a**) Comparison of the number of TFs regulating GO terms in humans and mice. (**b**) Proportion of orthologous TFs relative to the average number of TFs. The red line in (**a**) represents the average number of TFs regulating GO terms in humans and mice. The smooth red curve in (**b**) represents the predicted proportion of orthologous TFs regulating GO terms.

Human and mouse biological functions were found to be regulated by similar numbers of TFs (Figure 4a) but by different members of TF families (Figure 4b). GO terms [36] associated with a small number of TFs are mostly regulated by orthologous TFs. However, for GO terms regulated by many TFs (as many as 400, i.e., $\sim e^{6}$), the proportion of orthologous TFs is as small as 50%. We conducted GO and pathway enrichment analyses [46] on these two TF groups and their interacting genes (Supplementary Materials, Figure S6). Even though the numbers of isolated TFs and their interacting genes were much smaller than those of the other set of genes, their functional profiles were very similar regarding GO terms and pathways. This outcome indicates that the isolated TFs are not null-function, though their interaction with those functions may be weaker. Although the amount of functional change caused by the formation or loss of isolated TFs is small, the related phenotype is still affected. These TFs, especially the isolated ones, thus function through their formation or loss like multiple switches that open or close to generate a unique phenotype or a divergent function during speciation.

## 4. Discussion

Our TF-to-TF network is based on the STRING database, which collects protein–protein interactions based on several types of evidence (see Materials and Methods). Interactions with genes for which there is little information may be under-represented in the list. However, because of the large amount of human RNA-seq data, the co-expression data coverage is comprehensive. TFs can regulate gene expression, so if such regulation exists, it is likely to be detected by “conserved co-expression” in STRING. Evidence of co-expression and from high-throughput laboratory experiments may include unbiased information on the TF-with-protein interactions. We adopted the interactions when there was any evidence regarding the type of interaction; therefore, the isolation of TFs is likely to be real.

Our TF database was constructed by collecting sequences with DBD. Some proteins own DBD bud does not have regulatory function and some proteins have regulatory function but do not include sequences that are similar to known DBD domain. The number of functional annotations and DBDs are growing but these are still incomplete for now. The quality of the annotation of regulatory function varies among species. Therefore, our analysis of acquisition and loss of transcription factors may be affected by the variation of the quality of functional annotation. The analysis will become more solid as many well-annotated genomes across whole mammal species become available.

In recent years, studies of the C2H2 TF family and several other TF genes have revealed the evolution of TFs [9,10]. A relationship between TF sequence evolution and changes in DNA binding properties has also been found [47,48]. Reports showing that TFs are evolutionarily conserved were based primarily on TFs with known DNA-binding sequence specificities, whereas reports showing that TFs are evolutionarily variable always considered entire TF families. We therefore hypothesized that there is another type of TF that, along with well-studied TFs, contribute to overall TF evolution. Three factors have been proposed to explain how TF evolution has circumvented the problem of negative pleiotropy: (1) alternative splicing, (2) short linear motifs, and (3) simple sequence repeats [49]. Until now, however, the regulatory logic behind overall TF evolution remains unknown.

We found that one-third of TFs constitute a new TF type that is isolated in the human TF-to-TF network and that tends to be peripheral in the network of PPIs. These TFs have rarely been reported in previous human TF-to-TF network studies. The characteristics of isolated TFs are consistent with the protein characteristics related to lineage-specific phenotypes. Mutations of these isolated TFs are far less lethal than those of other TFs, indicating the high tolerance of the regulatory network to the evolution of these genes. The less strongly interacting genes encoding these isolated TFs contribute to less pleiotropic regulation. The other two-thirds of TFs make up a large connected TF component of the human TF-to-TF network containing nearly all TFs with known DNA-binding specificities.

Our comparative study of mammalian TFs presents an overview of TF member variation and demonstrates that TF evolution in mammals is ubiquitous—with changes observed in closely related species, not just between humans and mice. Starting from the same TFs in the shared common ancestor, the turnover of TFs during mammalian evolution and species–specific formation and loss events have gradually led to unique sets of TFs. In our human-mouse model, the overall force of TF formation and loss tends to be unilateral, with the overall expression level of interacting genes in a species being either relatively higher or lower. Changing the expression level of functional genes will consequently change phenotypes and pathway efficiency, an idea that is confirmed by the evidence in this study.

An isolated TF has a GO functional term overlay similar to that of connected TFs, which means that isolated TFs can also adjust a wide range of functions that are mainly regulated by connected TFs. We found that each GO term is regulated in humans and mice by a similar number of TFs, which are largely non-orthologous.

We believe that the gain and loss of TFs, mainly the isolated ones, is not a useless process, even though these changes are prevalent and tolerable to organisms. These changes will largely affect the properties of an interacting gene, such as its interaction and expression. When interacting TFs are absent or newly emerging, the same interacting genes will have different expression levels. As TF evolution has been frequent and widespread throughout mammalian history, large-scale phenotypes and pathway efficiencies have been shaped among species. These observations improve our understanding of the consequences of TF evolution.

We therefore hypothesized that these connected TFs follow the common TF regulatory pattern, with their conserved members possibly forming the backbone structure of the regulatory network. In contrast, the variable isolated TFs tune the flow of the regulatory network and give rise to species uniqueness by acting as on/off switches. This scenario explains how TFs can evolve while tolerating negative pleiotropic effects and identifies a major source of TF evolution and why TF numbers vary among species.

This situation may be best visualized by regarding the members of TF families as regulatory switches. During evolution, species may have modified the flow of the regulatory network by selecting different on/off states. Isolated TFs are an ideal tool for accomplishing this task: the relatively less lethal phenotypes of isolated TFs make them more tolerant to changes during speciation. In addition, emerging TFs in different species can diversify the expression profiles of their target genes, resulting in an adaptive phenotype for each species. Consequently, phenotypes have evolved by turning multiple switches on and off—in other words, through the formation and loss of isolated TFs.

## Figures and Tables

**Table 1:** Different features of large-component and isolated transcription factors (TFs).

TF Type	TF Number	TFs with Lethal Phenotype	TFs Absent in Mouse	Degrees
large component TFs	1040	507 (48.8%)	40 (3.8%)	77.9 ± 127.8
Isolated TFs	515	26 (5.0%)	189 (36.7%)	10.6 ± 8.8

Values were acquired by network analysis and TF annotation. Large-component TFs refer to the largest connected component in a TF-to-TF network. Isolated TFs comprise one four-TF component, 12 two-TF components, and other TFs with no TF–TF interactions. Degree indicates the average number of degrees of TFs in a human gene interaction network. The “lethal” phenotype was assigned to genes identified from a search using the keyword “lethal”.

**Table 2:** Mammalian phenotypes of representative genes that interact with KRAB-C2H2 and have low expression in humans.

Organ	Mouse Normal Phenotype	Gene with KRAB-C2H2 Interactions	Mammalian Phenotype	Mouse Knock-Out Phenotype	Human (Monkey) Normal Phenotype
Tail	Horizontal tail	CACNA1B	MP:0003382	Straub tail	(Vertical tail)
	Long tail	RIPK4	MP:0000592	Short tail	Without tail
Tooth	Continually growing teeth	OTUD7A	MP:0003053	Delayed tooth eruption	Permanent tooth
Hair	Fur-covered	CTSL2	MP:0000414	Alopecia	Hairless
		CTSL2	MP:0000417	Short hair	
Skin	Epidermis < 25 μm	CTSL2	MP:0001219	Thick epidermis	Epidermis > 50 μm
	Normal dermis	CTSL2	MP:0001245	Thick dermal layer	Thicker dermis than mouse

Organs exhibiting obvious phenotypic divergence between humans and mice are listed. Mammalian and mouse knock-out phenotypes were obtained from MGI. The phenotypes for all analyzed genes are listed in Supplementary Materials, Table S5.

## Supplementary Materials

The following are available online at https://www.mdpi.com/2073-4425/11/10/1148/s1. Table S1: Variation in TF family size among 96 mammalian species and human isolated TF list. (a) Number of TFs in TF families. (b) Isolated TF list and connected TF list in human. Table S2: Formation and loss events in 48 TF families. The number of gain events on branches and the number of loss events on branches. Table S3: Number of edges between different types of TFs in mouse and rat gene interaction networks. (a) TFs with DBD loss among mouse and rat. (b) TFs with gene loss among mouse and rat. (c) TFs without loss among mouse and rat. Table S4: Human and mouse gene expression data. RNA-seq data of 15,796 orthologous genes in cerebellum, heart, kidney, liver and testis. Table S5: Mammalian phenotypes of genes that interact with KRAB-C2H2 and have low expression in humans. Mammalian phenotypes of genes were obtained from MGI. Table S6: The effect of loss of SHOX in mouse inferred from the tail phenotypes associated with mutations in mouse FGFR3, the orthologous target gene of human SHOX. Figure S1: Human TF-to-TF network that shows interactions between transcription factors. Gray blocks are isolated TFs; the blue blocks are main-net TFs; lines are TF–TF interactions. Figure S2: Functional cartography of experimentally determined gene interactions. We characterized each gene in the human PPI network according to its within-module degree *z*-score (*z*) and participation coefficient (*p*). Genes were classified into eight groups: (NA) those with no experimental interactions, (R1) ultra-peripheral nodes (*z* < 2.5 and *p* < 0.05), (R2) peripheral nodes (*z* < 2.5 and 0.05 ≤ *p* < 0.625), (R3) non-hub connector nodes (*z* < 2.5 and 0.625 ≤ *p* < 0.8), (R4) non-hub kinless nodes (*z* < 2.5 and *p* ≥ 0.8), (R5) provincial hubs (*z* ≥ 2.5 and *p* < 0.3), (R6) connector hubs (*z* ≥ 2.5 and 0.3 ≤ *p* < 0.75), and (R7) kinless hubs (*p* ≥ 0.75). Figure S3: Total number of TFs in 96 mammalian species. The black bar is the total number of TFs in a species. The species trees are time trees from TimeTree. Figure S4: Shaping of species-specific phenotypes by species–specific TFs. The blue ellipse is orthologue TF and the orange ellipse is non-orthologue TF. Figure S5: Glycolytic pathway component ALDOA and its expression levels in humans and mice. (a) Initial steps of glycolytic pathway. White block: metabolite of pathway. Gray block: enzyme interacting with isolated TF. Orange block: enzyme interacting with non-orthologous TFs in human. (b) Expression of ALDOA. The blue bar is the expression of ALDOA in mouse. The orange bar is the expression of ALDOA in human. Figure S6: Enrichment analysis of gene ontology terms and pathways. (a) Enrichment analysis of gene ontology (GO) terms. X-axis: GO terms; Y-axis: percentage of genes in GO term. Orange bar: connected TFs and their interaction genes. Blue bar: isolated TFs and their interaction genes. (b) Enrichment analysis of pathways. X-axis: percentage of connected TFs and their interaction genes. Y-axis: percentage of isolated TFs and their interaction genes. Blue dot: pathway.
